# Fifth Metatarsal Base Fracture Combined With Fracture of the Os Peroneum

**DOI:** 10.5435/JAAOSGlobal-D-22-00172

**Published:** 2022-10-06

**Authors:** Zheng Dan Wang, Hui Li, Lin Li, Quan Yu Dong, Xiao Heng Ding

**Affiliations:** From the Department of Hand, Foot and Microsurgery, the Affiliated Hospital of Qingdao University, Qingdao City, Shandong Province, China.

## Abstract

Fracture of the os peroneum is rare, and displacement of the fracture can be indicative of a tear in the peroneal longus tendon. A fifth metatarsal base fracture is a common injury caused by sudden inversion and plantar flexion of the hindfoot. We observed a rare case of a fifth metatarsal base zone I fracture combined with a displaced os peroneum fracture in a 34-year-old woman. The patient was treated with resection of the os peroneum and repair of the peroneal longus tendon, as well as open reduction and internal fixation of the fifth metatarsal base. After exposing the fragment of the fifth metatarsal base, the distal part of the fractured os peroneum was found to be located just under the fracture site. There were no complications or discomfort of the foot or ankle at 2 years postoperatively. Resection of the os peroneum and direct repair of the peroneal longus tendon were easily performed after the fifth metatarsal base fragment was exposed. This was an innovative method for performing peroneal longus tendon repair in the deep portion of the midfoot.

The os peroneum is an accessory ossicle of the peroneal longus tendon that is located around the peroneal tunnel of the cuboid bone; it was reported to exist in 5% to 45% of individuals and be bilateral and symmetrical in most cases.^1,2,3,4,5^ A bipartite appearance is a frequent finding, occurring in approximately 30% of adults with an os peroneum injury.^6^ Os peroneum injury has been previously reported and can be caused by supination and plantar-flexion forces, which can cause tensile loading across the os peroneum.^7-9^ Treatment includes excision of the bone fragments with direct repair of the tendon and tenodesis of the peroneal longus to the brevis or cuboid.^10-12^ A fifth metatarsal base fracture is a common injury caused by sudden inversion and plantar flexion of the hindfoot, which generates tension at the insertion of the lateral band of the plantar aponeurosis.^13-15^ According to the fracture site, these fractures are classified into three zones. Zone I comprises the most proximal part of the tuberosity of the fifth metatarsal and includes the insertion of the peroneus brevis tendon, the peroneus tertius, and the lateral band of the plantar aponeurosis. Zone I fractures are the most common and tend to heal reliably with conservative treatment.^16-18^

These two fractures are commonly isolated and do not occur together. However, we encountered one case of a fifth metatarsal base zone I fracture combined with a displaced os peroneum fracture. To the best of our knowledge, no studies have previously reported this injury.

## Case Report

**Figure 1:**
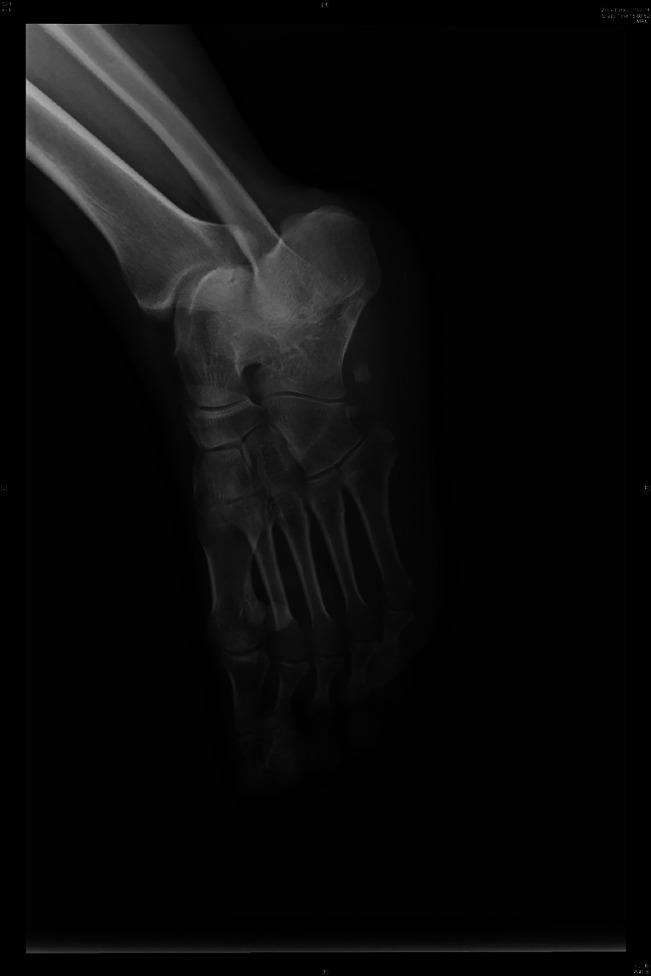
Preoperative radiograph showing a fifth metatarsal base fracture and displaced os peroneum fracture.

A 34-year-old healthy female patient presented to the emergency department. She reported falling down while riding an electrical bike one day prior and had sprained her ankle. Obvious swelling was observed on the lateral side of the midfoot. The patient could not bear weight because of pain, and she felt tenderness at the fifth metatarsal base and calcaneocuboid joint. There were no motor or sensory deficits in the foot or ankle. The radiographic views of the foot showed a fifth metatarsal base fracture in zone I and II os peroneum fragments (Figure 1). A three-dimensional CT scan was performed and showed that the os peroneum was fractured and displaced and that the proximal end had retracted under the calcaneus. The nonsclerotic, sharp margins and notable diastasis of the two fragments indicated a fracture rather than a bipartite variant of the os peroneum.

**Figure 2:**
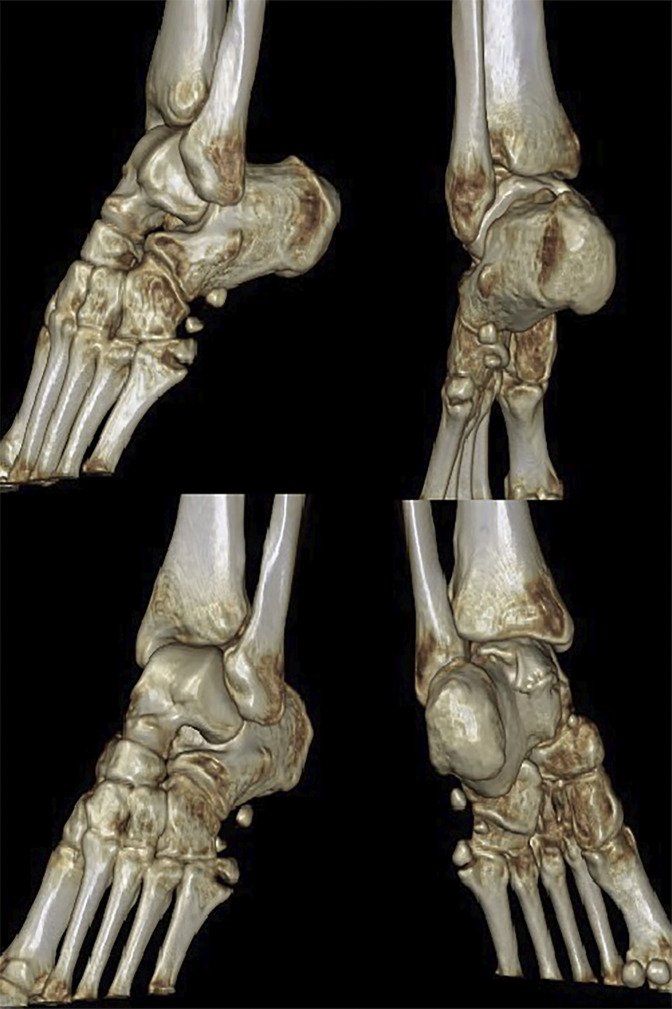
Three dimensional CT scan showing a fifth metatarsal base fracture and displaced os peroneum fracture.

In addition, the fifth metatarsal tuberosity was fractured, and the fracture has obvious displacement on CT scan (Figure 2). Therefore, the diagnosis was a peroneal longus tendon rupture with fractures of the os peroneum and fifth metatarsal base (zone I). The informed consent was obtained from the patient. Surgery was done 5 days after injury.

**Figure 3:**
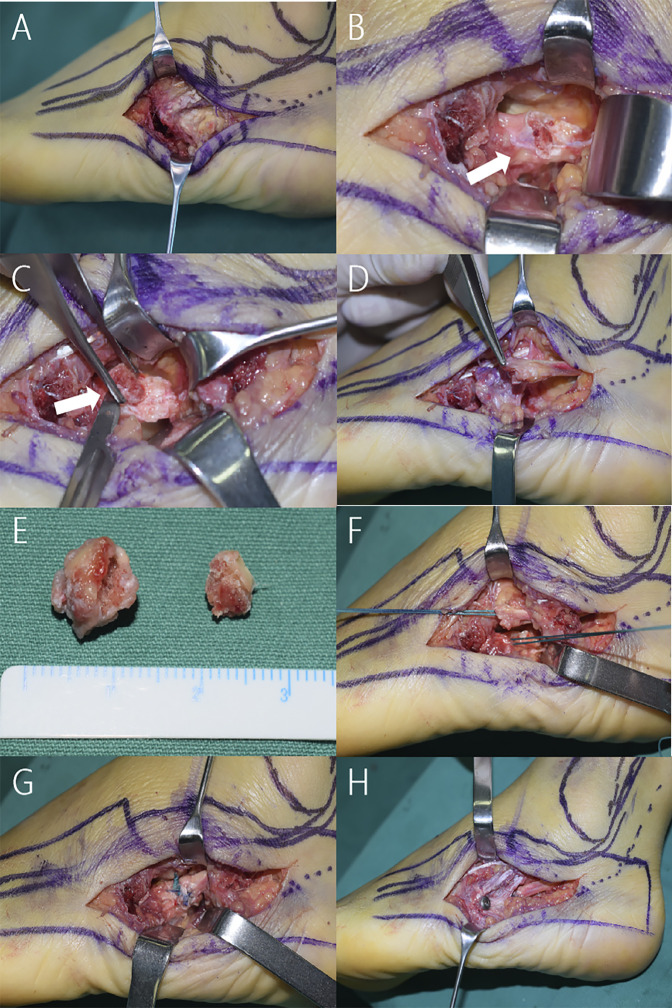
Photographs depicting the surgical procedure. **A**, The fracture of the fifth metatarsal base was revealed; **B** and **C**, white arrows showed the distal fragment of the peroneal longus tendon; **D**, the proximal fragment of the peroneal longus tendon; **E**, removed fragments; **F** and **G**, peroneal longus tendon sutured with a Kessler stitch; **H**, the fifth metatarsal base fracture fixed with one 3.5-mm cannulated screw and one 1.5-mm Kirschner wire.

After spinal anesthesia, resection of the os peroneum fragments and repair of the peroneal longus tendon, as well as open reduction and internal fixation of the fifth metatarsal base, were performed with the patient in the lateral position. A 6-cm longitudinal incision was made over the peroneal tendons centered on the plantar border of the cuboid. After soft-tissue dissection, the fracture of the fifth metatarsal base was exposed (Figure 3, A), and a proximal fragment of the os peroneum was found along the peroneal longus tendon tunnel. The tendon sheath around the peroneal brevis was released, a fragment of the fifth metatarsal base was exposed, and the distal part of the fractured os peroneum was found to be located just under the fracture site. We dissected and removed the fragment from the tendon, which was approximately 10 × 10 × 10 mm in size (Figure 3, B and C) and removed the proximal fragment, which was approximately 5 × 7 × 5 mm in size (Figure 3, D and E). The peroneal longus tendon was sutured with a Kessler stitch and reinforced with a 2 to 0 absorbable suture (Figure 3, F and G). We then reduced and fixed the fracture of the fifth metatarsal base with one 3.5-mm cannulated screw and one 1.5-mm Kirschner wire to prevent rotation of the fragment (Figure 3H).

After the operation, the ankle was immobilized with a short leg splint in a neutral position. Active range of motion was allowed at 6 weeks postoperatively. The patient was allowed to wear normal shoes and begin full weight-bearing at 10 weeks after surgery, and she was encouraged to begin balance training and proprioceptive exercises at 12 weeks postoperatively.

**Figure 4:**
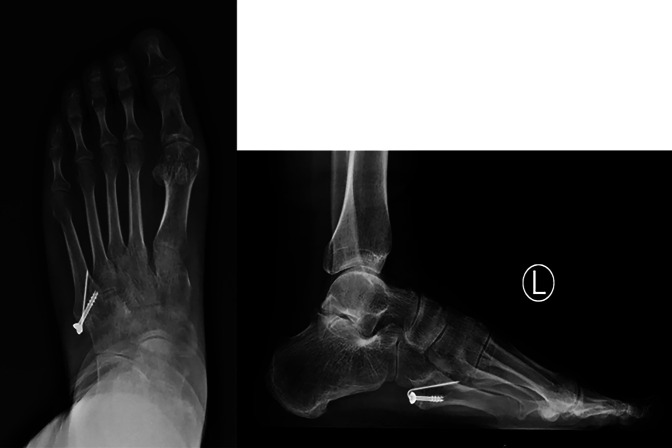
Postoperative 2 years radiographs.

There were no complications or discomfort of the foot or ankle at 2 year postoperatively, the American Orthopedic Foot and Ankle Society hindfoot-ankle score^19,20^ was 100 points, and the visual analog scale score was 0 points. At the last follow-up visit, the patient had radiographs taken and an ultrasonography to check the tendon. The results showed the fifth metatarsal base fracture union (Figure 4) and good continuity and thickening of the peroneal longus tendon, and the first metatarsal flexion power was normal compared with that of the contralateral side.

## Discussion

The os peroneum is a sesamoid of the peroneal longus tendon. In an anatomic study, it was found to be fully ossified in 20% of cases and not fully ossified in 75% of cases while in another anatomic study, the os peroneum was observed in 5% of cases.^21^ Additionally, the os peroneum is present bilaterally in 60% of affected patients.^2,22^ The os peroneum can be round or oval in shape, and a fracture of this bone can be easily confused with a bipartite variant of the os peroneum.^23^ The average size of the os peroneum is 4 mm in thickness and 13 mm in length. Approximately 30% of os peroneum bones are bipartite.^6^

Differentiating a bipartite os peroneum from an acute fracture of the os peroneum can be challenging. Generally, the distance between bipartite fragments is less than 2 mm, with smooth sclerotic margins; by contrast, irregular, nonsclerotic, sharp margins and a “puzzle piece” appearance indicate an acute fracture of the os peroneum.^1,6,24^ In our case, the distance between fragments was more than 10 mm, with nonsclerotic, sharp borders that conformed intraoperatively. Furthermore, displaced os peroneum fractures are indicative of complete rupture of the peroneal longus tendon.^25^ Avulsion fractures of the fifth metatarsal tuberosity usually occur in an inverted, plantar-flexed hindfoot when weight is placed on the plantar aponeurosis, the insertion of which is located at the base of the fifth metatarsal bone.^13-15,26,27^

The peroneus longus enables plantar flexion of the first ray, pronation of the forefoot, and eversion of the hindfoot. The first tarsometatarsal joint can be locked by peroneal longus contraction during standing. In addition, it is the first muscle to respond to a sudden ankle inversion sprain and passively stabilizes the ankle during inversion-supination of the hindfoot; the first metatarsal becomes locked in the plantar-flexion position to compensate for the retraction power of the peroneal longus, and the forefoot is in the pronation position compared with the hindfoot.^4,28,29^ Under rare conditions, fractures of the fifth metatarsal bone and os peroneum may occur together. In the current case, we suspected that the patient sprained her ankle during the fall from the electrical bike; the foot was plantar flexed, and the hindfoot was inverted, which caused the fifth metatarsal base to fracture. The forefoot should be pronated to compensate for the traction force; however, for some reason, it was supinated, and the peroneal longus tendon was pulled, which caused fracture of the os peroneum. To date, no studies have reported an os peroneum fracture combined with a fifth metatarsal base fracture, and more cases and biomechanical studies need to be conducted to support our postulation and verify this mechanism.

Several surgical techniques have been reported, including internal fracture fixation, fragment excision with tenodesis of the peroneal longus tendon to the brevis, and anchoring to the cuboid or calcaneus.^10,30,31^ Direct repair is quite difficult to perform because the distal stump of the tendon is often hidden under the cuboid notch in the deep midfoot.^32,33^ In the current case, by exposing the fifth metatarsal tuberosity and the insertion of the peroneal brevis tendon, we achieved better direct visualization of the distal stump of the peroneal longus tendon and distal pole of the os peroneum, which made it easier to achieve tendon reapproximation through end-to-end anastomosis. Therefore, in cases of an isolated os peroneum fracture or midfoot peroneal longus tendon rupture, we may consider performing a fifth metatarsal tuberosity osteotomy to expose the fracture fragment and the end of the tendon. This method is similar to performing a medial malleolar osteotomy for medial osteochondral lesions of the talus and an olecranon osteotomy for fractures of the humeral trochlea.

In conclusion, an os peroneum fracture is an uncommon type of lateral foot injury. We present a rare case of a fifth metatarsal base zone I fracture combined with a displaced os peroneum fracture, which indicated complete rupture of the peroneal longus tendon. The novel method applied, which facilitated treatment, consisted of resecting the os peroneum fragments and directly performing tendon anastomosis in the deep portion of the midfoot by osteotomizing the fifth metatarsal tuberosity and exposing the fracture fragment and peroneal brevis tendon.
